# A developmentally descriptive method for quantifying shape in gastropod shells

**DOI:** 10.1098/rsif.2019.0721

**Published:** 2020-02-12

**Authors:** J. Larsson, A. M. Westram, S. Bengmark, T. Lundh, R. K. Butlin

**Affiliations:** 1 Department of Animal and Plant Sciences, University of Sheffield, Sheffield, UK; 2 IST Austria, Klosterneuburg, Austria; 3 Mathematical Sciences, Chalmers University of Technology and University of Gothenburg, Gothenburg, Sweden; 4 Department of Marine Sciences, University of Gothenburg, Stömstad, Sweden

**Keywords:** growth, morphometrics, snail shells, shape variation

## Abstract

The growth of snail shells can be described by simple mathematical rules. Variation in a few parameters can explain much of the diversity of shell shapes seen in nature. However, empirical studies of gastropod shell shape variation typically use geometric morphometric approaches, which do not capture this growth pattern. We have developed a way to infer a set of developmentally descriptive shape parameters based on three-dimensional logarithmic helicospiral growth and using landmarks from two-dimensional shell images as input. We demonstrate the utility of this approach, and compare it to the geometric morphometric approach, using a large set of *Littorina saxatilis* shells in which locally adapted populations differ in shape. Our method can be modified easily to make it applicable to a wide range of shell forms, which would allow for investigations of the similarities and differences between and within many different species of gastropods.

## 1. Introduction

Snail shells are a beautiful example of how seemingly complex structures in nature can be described by simple mathematical rules. Logarithmic helicospirals, or conchospirals, are spirals that increase with a constant factor in height and radius for each revolution around a coiling axis, and they are well known to approximate the shell development of most gastropods [1–4]. Raup developed a method for describing self-similar shells by measuring a set of growth-related parameters and investigating the related shape space [5,6]. Several extensions have been made to Raup’s initial version [7–12], making it possible to model a more variable collection of shells and to give more accurate representations of features such as the aperture inclination. In addition to these fixed reference frame descriptions, there have also been efforts to describe the growth locally at the aperture, which describes the construction process from the viewpoint of the snail [13,14]. This type of method has rarely been used for quantification because it is difficult to infer the parameter values directly from empirical data, such as two-dimensional (2D) photographs, without first obtaining the parameters of a Raup-like description.

Despite the strong connection between these growth-related developmental parameters and the shell shape, population-level studies of shape variation have often favoured the more general method of landmark-based geometric morphometrics (GM) using the Procrustes method [15]. This approach quantifies the variation of a set of homologous points, called landmarks, positioned on images. It is widely and successfully used for morphological analysis of many biological organisms and structures, including snail shells [16–18]. However, it has some drawbacks when considering gastropod shells due to their spiralling accretionary construction process, where the shell grows by new material being deposited at the aperture. One issue with this process is that there is only one truly homologous point on the shells, the apex. The other points used are often semi-landmarks, points at arbitrary positions on curves where there is a lack of corresponding anatomical features. Another limitation is that the GM method does not provide a description directly relating to the shell’s development in the same way that a growth-based method does, making it harder to interpret the shape variation in biologically meaningful terms. Also, in GM analyses allometric changes can be hard to separate from other size-related variability.

There are examples of inferring growth parameters from three-dimensional (3D) μ-CT data [19]. However, this is both expensive and time-consuming, and thus not currently realistic for large sample sizes. None of the growth-based methods mentioned above have so far been implemented to quantify shape variation of large empirical datasets that includes variable aperture shapes. This is one of the reasons why GM is the current standard method despite lacking the direct connection to development. Therefore, we have developed a high-throughput method for quantification of shape variation in shells with variable aperture shapes using commonly available 2D data, which is built on the original ideas of Raup. This gives an intrinsic shape description of each shell in 3D, with developmentally descriptive parameters, i.e. parameters that can be clearly related to the accretionary growth of the snail shell. This will make it possible to relate the different aspects of shape to environmental and functional factors, and developmental processes. Additionally, since the parameters describe the shells intrinsically, we can extend the analysis by including more shells, e.g. from different sample sites or different species, and directly compare the distributions in the shape space. By contrast, GM analyses are specific to their datasets.

We have used the marine snail *Littorina saxatilis* to test our method because of its high shape variability, see [20] for a review of this species. In particular, we focused on the differences between two ecotypes, one adapted to resist crab predation by having a large, thick shell with a narrow aperture [21], and the other adapted to endure wave action and characterized by having a small shell with a round and relatively large aperture [22]. This ecotype dimorphism can be found on rocky shores throughout the north Atlantic coasts, and is especially well studied from the viewpoint of local adaptation, speciation and parallel evolution in parts of Spain, Sweden and the UK [23–26]. In this analysis, we investigated the Swedish system, and we have focused specifically on shape, which is one of the adaptive traits that differ between the ecotypes, and which has been shown to have a high heritability [27–29]. Some genetic differences between similar environments on geographically close islands (less than 10 km), have been observed in the Swedish system [30], thus it is possible that there are also phenotypic differences between sites at this scale. Therefore, we investigated how shell shape varies across boundaries between adjacent crab-type and a wave-type environments, and compared this pattern between separate sites.

Recent research on *L. saxatilis* has mainly used GM for quantifying shape [18,30], but other methods have also been used, including linear measurements [31], outline analysis [32], and a version of Raup’s original growth parameters [33]. Since all these methods can quantify shape variability, the way to choose which method to use should be decided by which type of description we are interested in [34]. GM makes it possible to quantify the ecotype variation, and to correlate this with changes in different parts of the environment and the genome [18]. However, with a more developmentally descriptive shape characterization it could be possible to get a clearer picture of which aspects of shape and growth are related to which biological and environmental factors, and to improve the understanding of which genomic regions underlie these differences. Similar advances could be made by applying this approach to other gastropods, other mollusc shells, or to other structures with similar growth patterns such as beaks or claws.

## 2. The model

**Figure 1.**
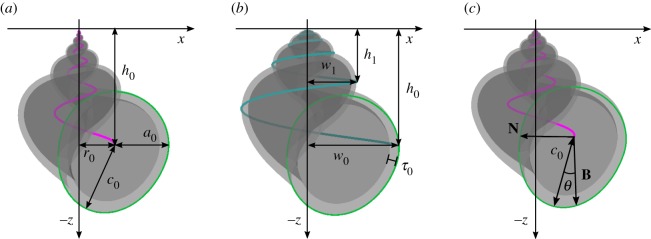
Two semi-transparent shell models, one concave (*g*_*w*_ > *g*_*h*_) on the left, one convex (*g*_*w*_ < *g*_*h*_) in the middle and one straight (*g*_*w*_ = *g*_*h*_) on the right, while all other parameter values are unchanged between them. In the examples on the left and right, the internal spiral *L*(*t*) are marked in pink, and on the middle one the external width spiral is displayed in teal. The growth parameters can be calculated as *g*_*w*_ = ln(*w*_0_/*w*_1_)/(2*π*) and *g*_*h*_ = ln(*h*_0_/*h*_1_)/(2*π*). The circliptic aperture is marked in green, it has the extension parameter *c* = *c*_0_/*a*_0_, and rotated by *θ* relative to the internal spiral’s normal plane defined by its normal **N**, and binormal **B**, here rescaled to reach the aperture curve. The relative thickness of the aperture is *τ* = *τ*_0_/*a*_0_. For the implementation, in this paper, all shells are normalized with respect to shell length, hence all linear measurement parameters are relative.

The model used in this analysis is based on an internal logarithmic helicospiral coiling around the vertical *z*-axis in 3D with apex at the origin [9,11]. We use separate growth parameters for the increase in width, *g*_*w*_, and height, *g*_*h*_, which relate directly to how much taller and wider the spiral becomes for each revolution around the coiling axis (figure 1). This internal spiral can be described in vector form by the equation

2.1 $$L(t)=(r_{0}e^{g_{w}t}\cos(t),\;-r_{0}e^{g_{w}t}\sin(t),\;-h_{0}e^{g_{h}t}),\;t\in [-2\pi n,0],$$

where *n* is the number of revolutions around the coiling axis to be included in the visualization, which can be chosen as a constant and should relate to the number of whorls visible for the species of interest. It is convenient to use start values *r*_0_ and *h*_0_ that are the radial and vertical distances from the origin to the spiral at the current aperture position, where *t* = 0. Since we are interested in spirals which are expanding downwards, as *t* increases to zero, in accordance with the standard way of visualizing snail shells, there is a minus sign in the vertical *z*-component. We only consider clockwise rotation downwards in this paper, it is, however, possible to change to anti-clockwise rotation by removing the minus sign in the *y*-component.

The growth parameters are assumed constant throughout the shell’s development. However, whenever *g*_*w*_ ≠ *g*_*h*_ there are allometric changes. If *g*_*w*_ = *g*_*h*_ we obtain a straight profile and therefore isometric growth, but if *g*_*w*_ > *g*_*h*_ then the shell will obtain a concave spire profile, and if *g*_*w*_ < *g*_*h*_ we get a convex profile (figure 1).

In order to include the variable aperture forms found in *L. saxatilis*, we introduce a one-parameter family of egg-like shapes that we have named ‘circlipses’, which smoothly combine a half-circle with a half-ellipse (examples in figures 1 and 3).

**Definition 2.1.**

A *circlipse* of size *a*_0_ with extension length *c*_0_, is defined by the radial function

2.2 $$C(s)=\left\{ \begin{array}{cc} \frac{a_{0}c_{0}}{\sqrt{c_{0}^{2}\cos^{2}(s)+a_{0}^{2}\sin^{2}(s)}}, & s\in [0,\pi )\;\\ a_{0},\; & s\in [\pi ,2\pi ), \end{array}\right.$$

around its reference point, i.e. the centre of the semicircle diameter.

The circlipse extreme point is at *s* = *π*/2 and has the value *C*(*π*/2) = *c*_0_. The extension parameter *c* = *c*_0_/*a*_0_ is the factor defining how much longer (or shorter) the major (or minor) semiaxis *c*_0_ of the ellipse is compared to the circle radius *a*_0_, where *c* = 1 gives a circle. This describes the directional eccentricity of the half-ellipse, and the value of *c* uniquely determines the circlipse up to size. This generating curve is assumed to not change shape during growth, however, the amount of the circlipse that is visible, and hence the resulting total aperture shape, might change over time, depending on the growth parameters *g*_*w*_ and *g*_*h*_.

The size of the aperture is also modelled to grow with a constant value for each revolution, hence we have an aperture growth function

2.3 $$A(t)=e^{g_{w}t},\;t\in [-2n\pi ,0],$$

where the aperture is assumed to increase the same value as the radial growth of the internal spiral *g*_*w*_. By only considering equal growth of the aperture and spiral radius, we restrict the shell shapes we can obtain to ones where the position of the aperture relative to the coiling axis does not change during growth, i.e. the radius of the spiral relative the total width of the aperture has the constant value *r*_0_/(*r*_0_ + *a*_0_) as the shell grows. This is a simplification needed in order to have a robust parameter approximation method given the currently available data. However, with improved input data it is possible that this assumption could be relaxed.

Using a circliptic aperture shape from equation (2.2) as a generating curve, sweeping out a surface as its reference point moves along the spiral defined in equation (2.1), we get the following surface function:

2.4 $$\begin{array}{rl} & S(t,s)=L(t)+A(t)C(s-\theta )(N(t)\cos(s)+B(t)\sin(s)),\\ & \;\left\{ \begin{array}{c} s\in [0,2\pi ),\;\\ t\in [-2n\pi ,0], \end{array}\right. \end{array}$$

where **N**(*t*) and **B**(*t*) are the unit normal and unit binormal for the internal spiral *L*(*t*). This gives an aperture plane which is oriented perpendicular to the curve, and has been suggested as a reasonable approximation of the true orientation for many shells [7]. We also allow the aperture circlipse to be rotated in this plane by the angular parameter *θ* around the reference point. Note that this angle has little to no effect on the shell shape if the aperture is close to circular, i.e. *c* ≈ 1.

By including a relative shell thickness parameter *τ* ∈ (0, 1) we can create an inner surface boundary which gives the model thickness without affecting the outside shape (figure 1). This is constructed by making a second surface with identical parameter values as the outside surface, except for the aperture size which will have the value *a*_0_(1 − *τ*), e.g. if the relative thickness is *τ* = 0.1 of the aperture size *a*_0_, then the internal surface will have aperture size 0.9*a*_0_.

The shell shape model presented above contains eight intrinsic parameters, *g*_*w*_, *g*_*h*_, *r*_0_, *h*_0_, *a*_0_, *c*, *θ* and *τ*, which is enough to create a large set of realistic shell shapes. Since they describe the accretionary construction process of the shells in nature, these parameters are straightforward to interpret in biological terms. The parameters are algebraically independent in the description above, but this is not the case after rescaling all shells to unit length, since for example spiral height *h*_0_ together with the elliptic extension length *c*_0_ = *a*_0_*c* are tightly linked with the total height. Shell size differs greatly between the analysed ecotypes, with crab-type shells generally being much larger than wave types. Normalization removes the part of the variation related to size. Rescaling, therefore, reduces the measured ecotype variability, and allows us to focus only on the shape variation. In addition to the parameters not all being algebraically independent, they are also unlikely to be biologically independent.

## 3. Sample collection and shell photography

We use snails collected from environmental contact zones on four islands within a few kilometres from each other on the Swedish west coast during 2013–2014: Ramsö (58°49′27.8″ N 11°03′45.3″ E), Inre Arsklovet (58°50′00.5″ N 11°08′19.6″ E), Ramsökalv (58°50′04.0″ N 11°02′26.5″ E) and Yttre Arsklovet (58°49′51.3″ N 11°07′59.0″ E), which are labelled CZA, CZB, CZC and CZD, respectively. For sites CZA, CZB and CZD, the snails are the same ones as in Westram *et al.* [35]. On each island, the snails were sampled across two environmental transitions in a transect going from an exposed cliff (wave) environment to a sheltered boulder field (crab) environment, and ending on another exposed cliff environment. This was done to include specimens from both ecotypes and intermediates from the environmental transition zones. The spatial position of each snail was recorded using a Total Station (Trimble M3), and simplified to a one-dimensional relative position along the shoreline by calculating a least cost path where cost is proportional to the inverse of local population density [35].

Approximately 600 snails were collected from each site, and four environmental factors describing the immediate surroundings were recorded along the sampling transect [35]. These factors were the type of substrate (bedrock versus boulders), presence/absence of barnacles (indicating wave exposure), presence/absence of fucoid seaweed (indicating a more sheltered environment) and local topography. They were combined into a single habitat score using a PCA, indicating the habitat type at each snail’s position.

**Figure 2.**
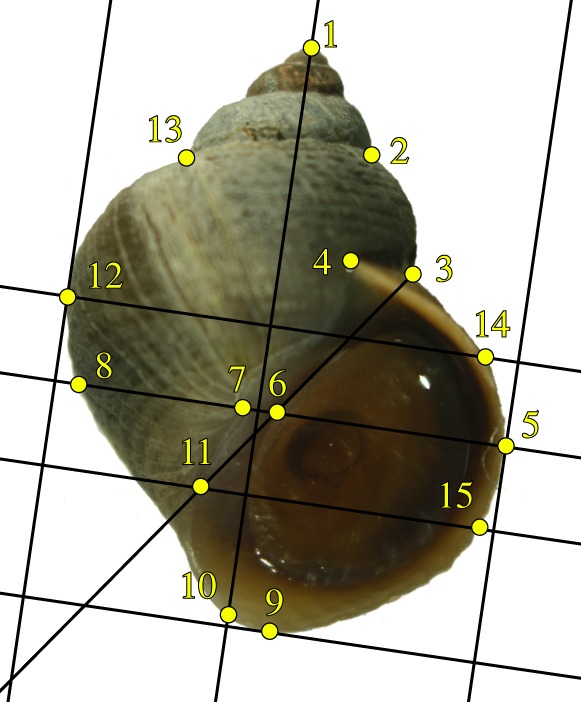
Landmarking procedure. The line from the apex, landmark 1, to the bottom of the shell, which is tangent to the empty part of the aperture defines the landmarking reference frame, we say that this line is ‘vertical’, and lines perpendicular to it are ‘horizontal’. The landmark point 10 is positioned at the lower extreme of the shell on this vertical line, and landmark 9 is at the lower extreme point of the whole shell. Landmarks 3, 13 and 2 are the three suture points on the outline where the most recently constructed consecutive whorls intersect, and landmark 4 is the end point of the suture at the current aperture. Landmarks 5 and 12 are the right and left extreme points of the shell in this reference frame, and using horizontal lines from these we define landmarks 8 and 14 as points on the opposite sides at the shell outline. On the vertical line through points 5 and 8, we position points 6 and 7 as the right and left points of the lip. A line from landmark 3 which is tangent to the empty part of the aperture is constructed, and landmark 11 is positioned where this line touches the outer edge of the lip, and landmark 15 is then positioned on the outer edge of the aperture using a horizontal line from landmark 11.

The shells were photographed in a standardized orientation using a digital camera, Canon EOS 1000D or 600D, mounted on a dissecting microscope and the positions of 15 points, *L*_1_, …, *L*_15_ (figure 2), were recorded for each image. These points were chosen for GM analysis and obtained according to a process similar to [30]. We will refer to these points as landmarks, for simplicity, although the majority are, in fact, semilandmarks. The shell thickness was calculated as the mean value of three measurements taken with a thickness gauge (Neoteck DTI Digital Dial Indicator Probe, 0.001 mm resolution) close to the current aperture at its widest point. The sex was recorded as either male, female or juvenile during dissection [36]. The juveniles were included in all analyses except for the comparisons between males and females. Specimens with missing data were excluded from the relevant analyses, making the total number at each stage at least 1923 shells.

## 4. Parameter approximation method

This method for estimating the parameter values for the shell shape model described in figure 1 has been implemented in Matlab.

### 4.1. Reorientation

In order to be consistent with the 3D coordinate description, we let the 2D image coordinates be *x* and *z*, and translate the coordinate system to have its origin at the apex point, i.e. *L*_1_ = (0, 0). We assume that the photo was taken such that the columnella is parallel with the viewing plane.

**Figure 3.**
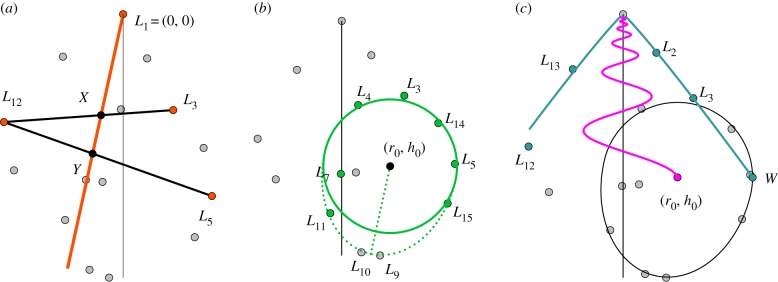
Procedure for approximation of parameter values. (*a*) Approximation of the coiling axis (orange) from the photograph, which is used for reorientation equation (4.1). (*b*) Position and size of the circular part of the aperture (green). The projection of the circliptic extension, and its orientation (dashed green). (*c*) Growth parameters generating the internal spiral, in pink, with the outer spiral profile in blue.

The landmarks *L*_3_, *L*_5_ and *L*_12_ are assumed to be placed at homologous positions on the last three half whorls, which allows us to find an approximation of the coiling axis by using properties of logarithmic helicospirals described in [37]. Applied to our set of known points, we use the following equations to approximate the orientation of the coiling axis:

4.1 $$\begin{array}{rl} X & =L_{12}+\alpha (L_{3}-L_{12}),\\ Y & =L_{5}+\alpha (L_{12}-L_{5})\\ \;\text{and}\;Y & =\beta X, \end{array}\}$$

where *X*, *Y* are the two unknown points where the coiling axis intersects the straight lines between the points on consecutive half whorls (figure 3*a*). We can use these equations since the widths of consecutive half-whorls are assumed to be proportional to each other, and since *X* and *Y* are on a straight line through the origin. We calculate the coordinates of *X* and *Y* by doing coordinate-wise algebraic manipulation of the above equations, resulting in a second degree polynomial in *α*. We solve this equation and choose the solution where *α* ∈ (0, 1), meaning that *X* and *Y* are restricted to being between their respective whorl points. The disregarded solution describes where *X* and *Y* lie on the extended lines through their whorl points, with the origin on the straight line between them. Using this, we can find the angle *v* needed to align the negative *z*-axis with the inferred coiling axis through *L*_1_, *X* and *Y*. After reorienting the landmarks to the desired coordinate system, we can proceed to approximate the values of the shape parameters.

### 4.2. Estimating the values

To approximate the aperture size and position in the above-defined coordinate system, we start by least square fitting a circle to the upper part of the aperture using *L*_7_, *L*_4_, *L*_3_, *L*_14_, *L*_5_, *L*_15_ and *L*_11_. This gives us the circlipse reference point and its size, i.e. the parameters *r*_0_, *h*_0_ and *a*_0_ (figure 3*b*).

To find approximations for the growth parameters, *g*_*w*_ and *g*_*h*_, we use the four landmarks *L*_2_, *L*_3_, *L*_12_, *L*_13_, together with the widest point of the fitted circle, *W* = (*r*_0_ + *a*_0_, *h*_0_). We use *W* rather than *L*_5_ as the widest point of the whorl since it relates to the reoriented coordinate system. However, these points are usually close together and so this choice is unlikely to make a large difference. The values are estimated by fitting exponential functions to the *x* and *z* coordinate values, respectively, as functions of *t*, and being a rotation of *π* apart. To make this approximation more robust we only consider functions close to the respective coordinate values of *W*, deviating with at most a factor of 0.01, since this point best satisfies our assumptions of being at the widest point of the whorl.

We also need to approximate the extreme point of the circlipse and its orientation, where we will take into account that the image is a projection of a 3D shape, and that the extreme point of the circlipse does not correspond to a specific landmark. We use both *L*_9_ and *L*_10_ to define the 2D projection of the circliptic extension and orientation; their mean length from the midpoint (*r*_0_, *h*_0_), $\hat{c}=(|L_{9}-(r_{0},h_{0})|+|L_{10}-(r_{0},h_{0})|)/2$, and mean angle relative to the *z*-axis, $\hat{\theta }=(\theta_{9}+\theta_{10})/2$ (dotted line in figure 3*b*).

We note that the aperture of the model is not parallel to the image *xz*-plane, as it lies in the normal plane of the spiral *L*(*t*) at *t* = 0, which can be found using the parameters previously obtained. To simplify the calculations, we rotate the curve *L*(*t*) around the vertical *z*-axis to make the aperture plane parallel to the *x*-axis, note however that the normal plane is still both tilted and rotated relative to the *xz*-plane. The angle between the spiral’s normal vector at the aperture, **N**(0), and the *x*-axis is, therefore, subtracted from the angle $\hat{\theta }$, giving us the desired approximation of the circlipse rotation angle *θ*.

We can now calculate what value of the circlipse extension length *c*_0_ is needed in order to have length $\hat{c}$ after projection. We calculate the length of the projected aperture unit vector in the direction of the extreme point, and use the fact that this vector has the same length relation before and after projection as the length *c*_0_ to the length $\hat{c}$ of its projection.

In addition to the seven parameters obtained from the landmark data, we have the thickness parameter obtained from separate measurements. Since we are interested in the relative thickness, we divide the measured thickness value for each shell with the approximated aperture size value *a*_0_, giving us the parameter *τ* = *τ*_0_/*a*_0_. To further remove size from this analysis, we normalize each shell to have unit length, defined as the distance between the apex and landmark *L*_9_. This only affects the value of the linear measurements in the model, *r*_0_, *h*_0_ and *a*_0_, while the rest of the parameters are relative, and hence invariant under scaling.

### 4.3. Assessing the approximation method

**Figure 4.**
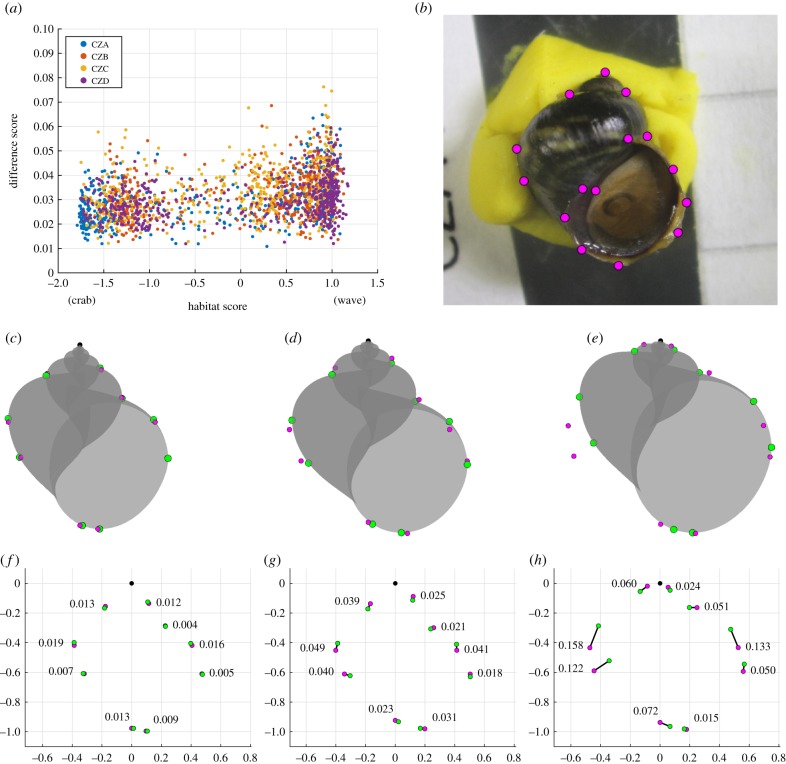
Success of the method. (*a*) The difference score for each model plotted against the habitat score, and coloured by site. (*b*) The original photo with superimposed landmarks of a typical example with a difference score of 0.032. We show the model with the best fit (*c*) with a difference of 0.011, the fit (*d*) of the typical shell in (*b*), and the worst fit (*h*) with a difference of 0.076. In the bottom row, we have the comparison between respective landmarks of the models above, numbers indicating the pairwise distance relative the shell height. Original landmarks are visualized in pink, and model landmarks are green.

To be able to tell if the parameter approximation method gives us reasonable shell models, we position points *M*_*i*_ on the models to mimic the original landmarks *L*_*i*_ on the photo. These points are then projected to the *xz*-plane to be compared with their respective original landmark points. However, only 10 of the original 15 points can be positioned on the models, and the apex is not included in the comparison since it by definition has the same coordinates for both sets of landmarks. Hence, only nine points are compared (figure 4*h*).

To reorient the shell, we start by rotating it around its coiling axis to get the aperture parallel with the *x*-axis, using the same angle as in the aperture parameter approximation. For the next step, we need to rotate the shell around the *y*-axis, i.e. in the 2D image plane, to get the same reference frame as when the original landmarking was done. We need to take into account both the reorientation angle *v* of the inferred coiling axis relative to the image, and the reference frame used in the original landmarking procedure, defined by the line between *L*_1_ and *L*_10_. Note that the apex stays fixed in the same position during the rotations since it is at the origin. After these rotations, we position *M*_10_ as the lowest point on the shell for which *x* = 0.

The points *M*_5_ and *M*_12_ are positioned at the widest points of the shell, i.e. maximum and minimum *x*-value of the shell’s the outline, and the points *M*_8_ and *M*_14_ are placed to have the same *z*-values but positioned on the outline on their respective opposite sides. The points *M*_2_, *M*_3_ and *M*_13_ can be found where the outlines of consecutive whorls have equal *x*- and *z*-values. Lastly, we put *M*_9_ as the extreme point in *z*-value.

We make an orthogonal projection to the *xz*-plane which gives the 2D coordinates to compare with the original landmark points. The difference score is defined as the mean distance between the nine pairs of corresponding points (figure 4*h*). We use the score obtained for the shells in this analysis to quantify the performance of the parameter approximation method. This is only a rough estimate of their likeness since the comparison relies on only nine points of the shells outline. Note also that this does not directly measure how accurate the parameter values are, but how well the model and original landmarks match.

## 5. Statistical analysis

### 5.1. Parameter analysis for the growth-based method

The growth parameters *g*_*w*_ and *g*_*h*_, and the circlipse extension parameter *c* were log-transformed before the statistical analyses.

To investigate how strongly the parameters were related to the habitat difference, we computed the Pearson correlation coefficient between each of them and the habitat score. This was done for each of the four sites separately and compared to see if the correlations were consistent or differed between them.

To visualize how and where the parameter values changed in transects across the environmental transitions, we rescaled the values of the parameters between [0, 1] at each site, and reoriented them such that greater values were associated with the wave habitat. Then we calculated a moving average using 10% of the total number of snails as a function of their position on the shoreline. This smoothed function was then viewed together with the habitat score. One growth parameter value outlier (figure 6) was removed for this analysis to make the rescaling consistent.

To investigate the presence of sexual dimorphism, we computed the canonical variable maximizing the differences between the sexes, a linear combination of the parameters, and compared the difference in distributions for males and females. We also calculated the correlation coefficients for each shape parameter with sex. The parameter with the strongest correlation was further investigated and viewed as a function of shore position, including the moving averages using 15% of the snails for each sex. This was done to examine whether the sex difference varied between the environments.

### 5.2. Geometric morphometrics

Using the same set of 15 landmarks as for the growth-based method, we investigated the shape variation using the traditional GM method implemented in the R package geomorph [38]. We conducted a PCA of the full set of shells to verify that we obtain results consistent with previous analyses, i.e. that the largest component of shape variation, PC1, relates to the difference in habitat. We also did a PCA of the parameters from the growth-based method, and calculated the correlation coefficient between the first PC of each method, together with visualizations of their associated shape variations, and used that as an indication of how well these two shape scoring systems coincide.

## 6. Results

### 6.1. Method assessment

The difference in landmark position between the original image and the model suggests that our method achieved a reasonable model approximation for most shells. All shells obtained a mean distance between landmarks on the original image and model of less than 0.076, i.e. 7.6% of shell length, and 96% of shells had a mean distance of less than 0.05 (figure 4). The most common mismatches between landmarks were in the vertical position of the two leftmost and two rightmost points (figure 4*h*), this can usually be attributed to an underestimation of the aperture size *a*_0_ when landmarks *L*_15_ and *L*_11_ are high up and close to landmarks *L*_5_ and *L*_7_, respectively. This is a result of the variability in landmark *L*_11_ when placed according to the landmarking procedure. The method was in general slightly more stable for crab-type shells (figure 4*a*), this could be because some wave-type shells did not have much spire visible, which can introduce some uncertainty of the position of the apex landmark, and this also causes the suture landmarks *L*_2_, *L*_3_ and *L*_13_ to conform less well to the assumptions of the new method. Since these are problems relating to the landmarking process in itself, it is also an issue for the GM method, and emphasizes the problems of not having true homologous points to work with on snail shells.

### 6.2. Comparison with geometric morphometrics

**Figure 5.**
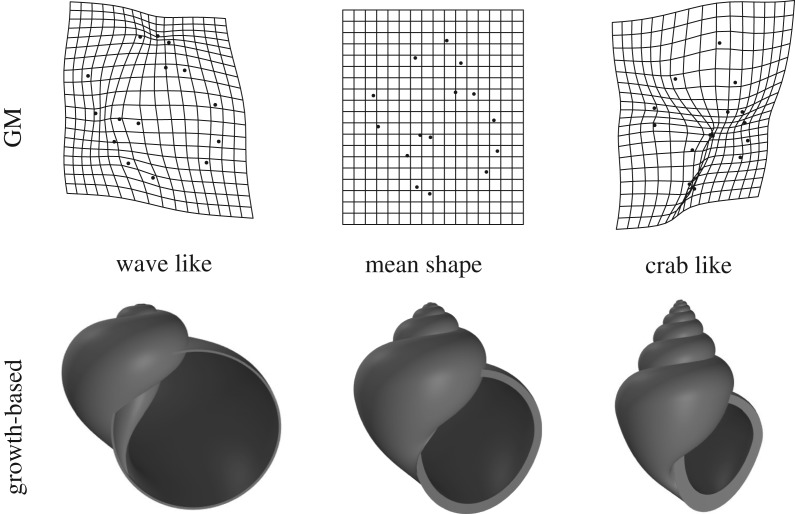
Visualizations of the means and extremes of PC1 for the geometric morphometrics method and growth-based method.

The PC1 scores from GM and the growth-based method had a high correlation with each other. The calculated Pearson’s correlation coefficient was *r* = 0.94. Visually, the two methods showed the same type of general shape changes when comparing the deformation grids of GM to the models of the growth-based method (figure 5). This variation is also consistent with the previously described shape differences between the habitats: small, narrow apertures and tall spires in the crab habitat, and large, round apertures with short spires in the wave habitat. For our new growth-based method, the habitat-related PC1 explained 53% of the total variation of the eight parameters.

### Growth-based method

**Figure 6.**
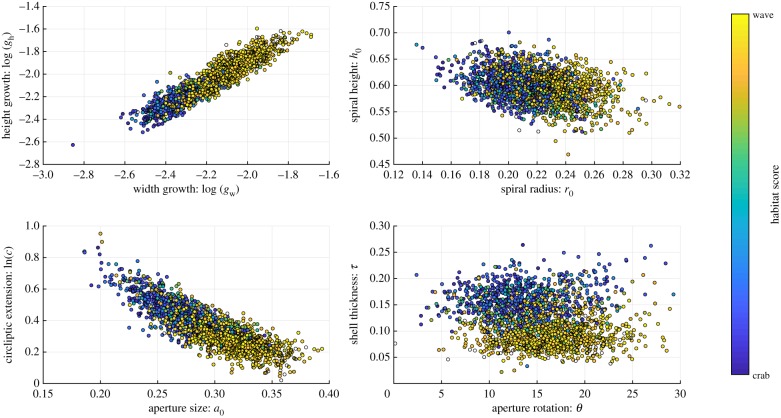
Distribution in the parameter space of the analysed shells, coloured by the habitat score.

In terms of ecotype difference, six of the eight parameters co-varied with habitat at all four sites, having moderate or high correlation coefficients (|*r*| > 0.45) at each site (table 1). The parameters that did not show a consistent correlation with habitat were the relative height of the spiral, *h*_0_, and the aperture angle, *θ*. The values of the six environmentally correlated parameters varied continuously between the habitats rather than splitting the snails into two separate clusters (figure 6), indicating that no intermediate shapes were missing. There was also substantial variation within the different environments, but this was smaller than between the habitats.

**Table 1.** The correlation coefficients for each parameter with the habitat score, separated by site. Positive correlation values indicate that larger values of that parameter were associated with the wave habitat, negative values indicate larger values were associated with the crab habitat.

	*g*_*w*_	*g*_*h*_	*r*_0_	*h*_0_	*a*_0_	*c*	*θ*	*τ*
CZA	0.763	0.758	0.485	−0.108	0.742	−0.65	0.419	−0.761
CZB	0.635	0.633	0.451	−0.19	0.582	−0.458	0.232	−0.714
CZC	0.717	0.69	0.515	−0.0341	0.619	−0.537	0.405	−0.689
CZD	0.735	0.711	0.477	−0.222	0.668	−0.523	0.0562	−0.641

**Figure 7.**
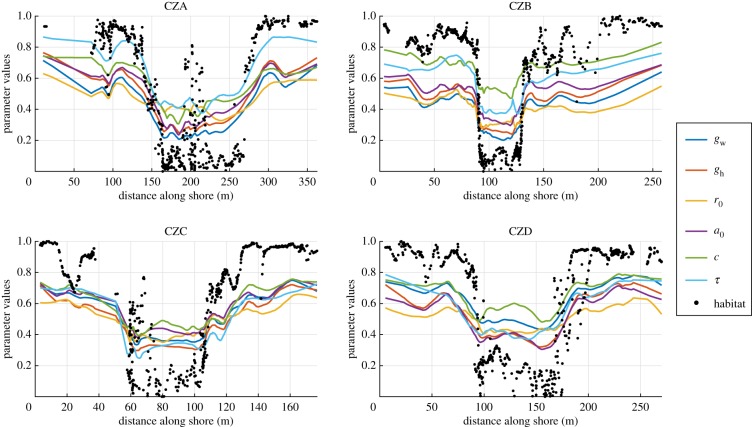
The relative variation of the parameters as a function of shore position, separated by site. Each line represents the moving average of one parameter, and the dots represent the habitat score at the position of each snail. Oriented such that the higher values are associated with the wave habitat. The two parameters *h*_0_ and *θ* were excluded from this figure since they had no clear correlation with habitat.

The six consistently habitat-correlated parameters covaried as the environment changed, and the main shifts in values were close to the environmental transitions (figure 7). Small areas of wave-type environment in the crab habitat, as in site CZA, did not have a great influence on the parameter values, while small crab-type environmental patches in the wave habitat showed a stronger effect on shape, as in site CZB. This has been observed before, and has been suggested to be an effect of crab predation being a stronger selective pressure than wave exposure [18]. In addition to the parameters covarying across the largest environmental transitions, we can also see that they covary to a large extent even within the separate environments. Note also that the independently measured thickness parameter shows a similar pattern to the other habitat-related parameters.

**Figure 8.**
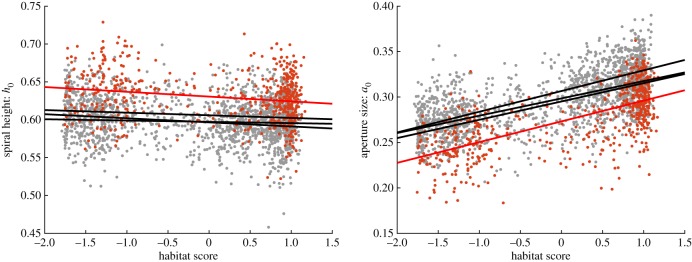
The difference in values of the spiral height *h*_0_ and aperture size *a*_0_ between sites. Site CZD in red, other sites in grey. Lines show the least square fits relative to the habitat score, one for each site with CZD in red.

At site CZD, we obtained a difference in some parameter values compared with the other sites. The parameters mainly showed the same type of variation relating to habitat (table 1), but located around a different mean value. This can be seen, for example, in the parameter with the lowest habitat correlation, the spiral height *h*_0_, as well as in a parameter with much stronger habitat correlation, the aperture size *a*_0_ (figure 8). The aperture rotation angle *θ* on the other hand, did not show this pattern, instead it changed to having a even weaker correlation with habitat at CZD compared with the other three sites (table 1).

**Figure 9.**
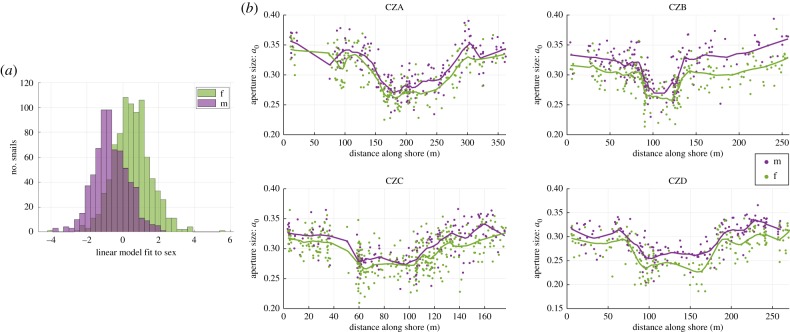
(*a*) Distribution of the sexes along the canonical variable maximizing their difference in means when combining the data from all sites. (*b*) The aperture size values *a*_0_ plotted as points along the shoreline with males in purple and females in green, juveniles not shown. Moving averages are plotted as curves of their respective colour.

There was also a difference in parameter values between males and females, independently of sites and habitat. Viewed along the canonical vector maximizing the distance between males and females from all sites combined, there was a clear difference between their means (1.4 s.d.), but the distributions were still mostly overlapping (figure 9). The parameter most strongly correlated with sex was the aperture size, *a*_0_, with males having larger apertures for their height than females (table 2). This, together with larger growth parameter *g*_*w*_ and *g*_*h*_, and smaller height, *h*_0_, and radius, *r*_0_, of the internal spiral, and smaller circlipse extensions, *c*, suggests a larger and rounder aperture, without changing the total width much. However, the difference between males and females was small compared with the total variation, and therefore the correlation was not very strong for any of the parameters, although it was fairly consistent in both types of habitats and at all sites (figure 9).

**Table 2.** Correlation coefficients for each of the parameters and sex, at each site. Positive correlation coefficients indicate that larger parameter values are associated with males, while negative correlations indicate larger parameter values are associated with females.

	*g*_*w*_	*g*_*h*_	*r*_0_	*h*_0_	*a*_0_	*c*	*θ*	*τ*
CZA	0.101	0.0754	−0.204	−0.292	0.146	0.0622	−0.0481	0.078
CZB	0.274	0.197	−0.107	−0.294	0.288	−0.147	−0.223	0.125
CZC	0.143	0.147	−0.21	−0.0964	0.183	−0.107	−0.173	0.137
CZD	0.183	0.144	−0.127	−0.207	0.24	−0.126	−0.147	0.072

## 7. Discussion

This new method for quantification and description of gastropod shell shape variation achieves reasonably accurate approximations despite using only 2D data designed for GM analysis. Note however that the accuracy is not measured for the individual parameters, but for how well the landmarks on the shell model that they generate coincide with the landmarks from the original image. The two main advantages to using a growth-based method over GM are that it describes the developmental process underlying formation of the shell structure, and that this description is intrinsic and not relative, meaning that different samples or species can be added to, and compared directly in the resulting parameter space. Having a growth-based description should give new insights into the environmental and genetic factors underlying variation in different aspects of shell shape. Additionally, the method generates shell models that can be used for further analyses, e.g. fluid dynamic studies of shells in water flows or structural analysis of shell strength, which relate back to the contrasting natural selection pressures for the ecotypes of *L. saxatilis* discussed here. It should be noted, however, that the models do not include any surface roughness or information on material strength or thickness variation. This needs to be taken into account in any further analysis.

This method provides an intuitive way of describing the shell shape variation of many gastropods. It is possible to apply this method to any structure which can be approximated as a tube with a circliptic cross section and which is increasing in size proportionally to, and along a, logarithmic helicospiral. This regular growth pattern is commonly, but not exclusively, found in snail shells, and is what allows us to go from a single 2D image to a 3D representation, which is not possible to do in general. Furthermore, the stability of finding the reference point of the aperture circlipse from its circular part, together with the flexibility of extending parts of it without affecting this reference point, is a feature which lets us apply this method to a large range of snail species (electronic supplementary material, appendix A). This idea could also be built upon, to account for an even more diverse range of shell shapes, by incorporating more complex aperture shapes as long as we can consistently fit a circle to part of the aperture. From this description, it should also be possible to convert the parameters to those of a growing tube model [14], giving us two different characterizations describing the same growth. This could further improve the understanding of shape variation from the perspective of the local accretion process at the aperture.

Using this method, we can account for certain types of variation in shape during growth. If the growth parameters are equal for a shell, *g*_*h*_ = *g*_*w*_, then it has isometric growth, i.e. the shape does not change over time. However, for this sample, we mainly obtained larger growth values for height than for width, *g*_*h*_ > *g*_*w*_, although still close to equal, suggesting a slightly convex spire profile (figure 1). Previous work has already shown some evidence of shape variation of *L. saxatilis* during growth using other methods [39–41], but using GM it can be hard to separate ontogenetic changes from other size-related variation [42]. To investigate how much size-related variation can be accounted for by the convexity described above, rather than, for example, changes in the growth parameters, further growth-based analysis of shells at different stages of development will be needed. For shells where allometry can be attributed to unequal but constant growth parameters, it is possible to use this method to visualize the ontogeny of a given shell, and to predict the future shape of a shell that will continue growing. For isometrically growing shells, this is trivial since their shape does not change over time.

In our analysis of *L. saxatilis* shells, we could quantify the same major differences between ecotypes which has been described in previous studies using other methods [22,30]. However, the variation described when using GM is interpreted by visual inspection of the point variation in thin plate splines obtained after a PCA, which therefore depends on the samples used. By contrast, in the growth-based analysis variation is described by a set of intrinsic values which are directly comparable between studies and gives a quantification of parameters such as growth rates. In addition, the description presented in this paper allows us to relate the current shape of the shell to how it developed over time. We obtained larger growth values in snails of the wave ecotype, meaning that their shells increase in height and width more per revolution than in the crab ecotype, and therefore the aperture and most recent whorl make up a larger proportion of the whole shell. The apertures were smaller in the crab ecotype but also more elongated. The reason for the relative spiral height *h*_0_ not varying much between habitats is that the elongation of apertures in the crab ecotype covaries with taller shell spires. The aperture rotation angle *θ* does not affect the shape of circular apertures and is therefore not informative in the wave habitat. To further understand how the correlation between parameters relates to constructional, environmental and genetic factors, more analysis is needed.

In addition to the large ecotype-related variation, we also found a consistent difference between the two sexes at all four sites, though the total effect this has on shape is very small. Some shape differences between the sexes have been detected in previous studies, although they were only described separately in terms of allometry at different growth stages for different habitats [29]. The differences found in this analysis mainly suggest that males have a slightly larger and rounder aperture relative to their size than females. This difference could be due to the position of their reproductive organs. Since the distributions are mostly overlapping, it is unlikely to be directly useful as a method for sexing individuals. However, the ability to pick up such a small difference and describe it in terms of growth could still be useful in future analyses and the model could be extended to consider the impact on internal volume.

We also found that the shape of snails at site CZD was consistently different from the other sites. This was mainly due to a difference in the position of the landmark *L*_4_, which is therefore also detectable as a difference when using GM. There are two possible explanations for this deviation: either the shells were consistently positioned differently for the photographs at this site, or there is a true difference in shape at that site. The shells were destroyed during dissection, and therefore cannot be examined further. Either way, there is a difference in landmark position on the photographs. If this is not a true shape difference, it suggests that changing to a more stable method of positioning shells and extracting data than the current method would be desirable. This highlights the problems of consistency in positioning and selection of homologous points on a structure that grows by accretion, a problem common to GM and our approach. However, the combined effect of variability in shell orientation and landmark position was small enough that it did not obscure the main shape variation of biological interest, the difference between ecotypes.

The method could be improved further. As noted before we could improve the input data, selecting different points and other geometric structures (e.g. manually placing the circlipse) in the images, and making use of outline data, as well as standardizing the shell position differently to be more optimal for finding growth parameters, for example, following the procedure found in [43]. This could improve both the accuracy itself, and the ability to measure the accuracy, and possibly lead to an automatization of the process. In addition, this could make it possible to compare a larger range of shell types, for example by allowing relaxation of the assumption that the spiral radius and aperture growth rates are equal. A slightly modified version of the parameter approximation method was applied to shells from other species of snails to illustrate its potential range of applicability (see electronic supplementary material, appendix A). Future effort will include making this method accessible to conchologists, without requiring full mathematical understanding of the procedure.

## Supplementary Material

Supplementary Information

## Data accessibility

The data used in this paper can be found at https://doi.org/10.15131/shef.data.9944462.v1.
