# Supplementary material for: A developmentally descriptive method for quantifying shape in gastropod shells
Source: J R Soc Interface. 2020 Feb 12;17(163):20190721. doi: 10.1098/rsif.2019.0721 (PMC7061706; doi:10.1098/rsif.2019.0721)
Supplement: Supplementary Information [file rsif20190721supp1.pdf]

A Supplementary material

By making two small modifications to the shell model presented above, we can apply it to a greater range of gastropod shell shapes. The first modification is adding the ability to remove a section from the aperture and only include the part that is actually constructed. Secondly, by calculating the aperture plane using the normal plane of a spiral tracing the widest part of the shell instead of the internal spiral, it is possible to reduce the variability of the aperture orientation that occurs when the internal spiral is very close to the coiling axis, i.e. r_0 is very small. These modifications have been included in a package, ShellShaper, that allows interactive fitting of the growth model to a shell photograph without landmarks, which is freely available at `https://figshare.shef.ac.uk/articles/ShellShaper/9944591`. Examples of models obtained using ShellShaper are given in figure 10. Note however that thickness is not measured from the images, and has been set to a standard value as it does not affect the outside shape. Further extensions could be to include more diverse aperture shapes, such as ridges, by modifying the generating curve rather than restricting it to a circlipse, or colour variation, such as the placement of bands.

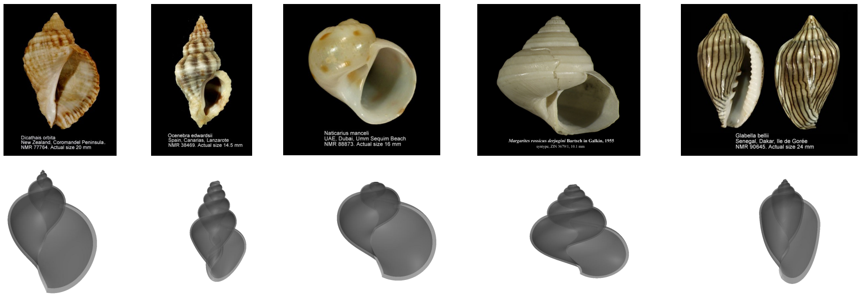

Figure 10: Examples of gastropod species which can be described using a slight variation of the model in section 2. Photographs obtained from the WoRMS database [43]. From left: *Dicathais orbita, Ocenebra edwardsii, Naticarius manceli, Margarites rossicus derjugini, Glabella bellii*
